# Population genomics reveals the expansion of highly inbred *Plasmodium vivax* lineages in the main malaria hotspot of Brazil

**DOI:** 10.1371/journal.pntd.0008808

**Published:** 2020-10-28

**Authors:** Thaís Crippa de Oliveira, Rodrigo M. Corder, Angela Early, Priscila T. Rodrigues, Simone Ladeia-Andrade, João Marcelo P. Alves, Daniel E. Neafsey, Marcelo U. Ferreira

**Affiliations:** 1 Department of Parasitology, Institute of Biomedical Sciences, University of São Paulo, São Paulo, Brazil; 2 Infectious Disease and Microbiome Program, Broad Institute of MIT and Harvard, Cambridge, Massachusetts, United States of America; 3 Departament of Immunology and Infectious Diseases, Harvard T. H. Chan School of Public Health, Boston, Massachusetts, United States of America; 4 Laboratory of Parasitic Diseases, Oswaldo Cruz Institute, Fiocruz, Rio de Janeiro, Brazil; University of Texas Southwestern Medical School, UNITED STATES

## Abstract

**Background:**

*Plasmodium vivax* is a neglected human malaria parasite that causes significant morbidity in the Americas, the Middle East, Asia, and the Western Pacific. Population genomic approaches remain little explored to map local and regional transmission pathways of *P*. *vivax* across the main endemic sites in the Americas, where great progress has been made towards malaria elimination over the past decades.

**Methodology/Principal findings:**

We analyze 38 patient-derived *P*. *vivax* genome sequences from Mâncio Lima (ML)–the Amazonian malaria hotspot next to the Brazil-Peru border—and 24 sequences from two other sites in Acre State, Brazil, a country that contributes 23% of malaria cases in the Americas. We show that the *P*. *vivax* population of ML is genetically diverse (π = 4.7 × 10^−4^), with a high polymorphism particularly in genes encoding proteins putatively involved in red blood cell invasion. Paradoxically, however, parasites display strong genome-wide linkage disequilibrium, being fragmented into discrete lineages that are remarkably stable across time and space, with only occasional recombination between them. Using identity-by-descent approaches, we identified a large cluster of closely related sequences that comprises 16 of 38 genomes sampled in ML over 26 months. Importantly, we found significant ancestry sharing between parasites at a large geographic distance, consistent with substantial gene flow between regional *P*. *vivax* populations.

**Conclusions/Significance:**

We have characterized the sustained expansion of highly inbred *P*. *vivax* lineages in a malaria hotspot that can seed regional transmission. Potential source populations in hotspots represent a priority target for malaria elimination in the Amazon.

## Authors’ summary

*Plasmodium vivax* is the geographically most widespread human malaria parasite and causes 80% of the malaria burden in the Americas. Here we use whole-genome sequencing to explore levels of parasite relatedness and infer *P*. *vivax* transmission networks in the upper Juruá Valley, the main transmission hotspot in Amazonian Brazil. We characterize a genetically diverse population that displays significant linkage disequilibrium, consistent with the local circulation of highly inbred but genetically distant parasite lineages. Noteworthy, these discrete lineages remain stable over time and share recent ancestry with parasites at a large geographic distance. These results illustrate the power of genomic epidemiology approaches to map potential source parasite populations and prioritize areas for targeted control interventions to eliminate residual *P*. *vivax* transmission in the Amazon and similar endemic settings worldwide.

## Introduction

*Plasmodium vivax*, the most geographically widespread human malaria parasite, causes significant morbidity in Central and South America, the Middle East, Central, South, and Southeast Asia, and the Western Pacific. Nearly 3.3 billion people are at risk of infection worldwide, with 14.3 million clinical vivax malaria cases estimated to occur each year [1]. *Plasmodium vivax* accounts for 80% of the approximately 900,000 malaria infections reported yearly in the Americas [2] and appears to be more resilient than *P*. *falciparum* to current control and elimination strategies [3].

Population genomic studies of *P*. *vivax* have revealed a surprisingly high genetic diversity within geographic populations in the Americas, the last continent to be colonized by humans, which is comparable to that in areas with substantially more intense malaria transmission in Southeast Asia. Nevertheless, there is only moderate differentiation across *P*. *vivax* populations from different countries [4–6]. The genetically diverse American populations of *P*. *vivax* are thought to have resulted from successive migratory waves and subsequent local admixture between parasites of different geographic origins [4,6]. Although whole-genome sequencing has been extensively used to measure parasite relatedness as a correlate of recent gene flow of *P*. *falciparum* in pre-elimination settings in Africa [7–10] and Southeast Asia [11,12], genome-wide variation remains little explored to map local and regional routes of *P*. *vivax* circulation in the Americas, a key information for designing malaria elimination policies [13].

Here we leverage genetic diversity and shared ancestry patterns to gain insights into *P*. *vivax* transmission in Brazil, a country that contributes 23% of malaria cases in the Americas. Although the countrywide incidence of malaria has dramatically decreased over the past decades, residual transmission remains entrenched in the Amazon Basin, where nearly 90% of the infections are due to *P*. *vivax* [14]. We analyze genome sequences from *P*. *vivax* isolates circulating in the Amazonian hotspot next to the Brazil-Peru border that accounted for 18% of the 218,000 cases malaria cases recorded in the country in 2018 [2]. We identify the sustained transmission of discrete *P*. *vivax* lineages throughout this hotspot, which must be considered by regional malaria elimination efforts.

## Methods

### Ethics statement

Study protocols were approved by the Institutional Review Board of the Institute of Biomedical Sciences, University of São Paulo and the National Committee on Ethics in Research of the Ministry of Health of Brazil (CAAE number, 6467416.6.0000.5467). Written informed consent was obtained from all study participants or their parents or legal guardians.

### Study site

**Fig 1:**
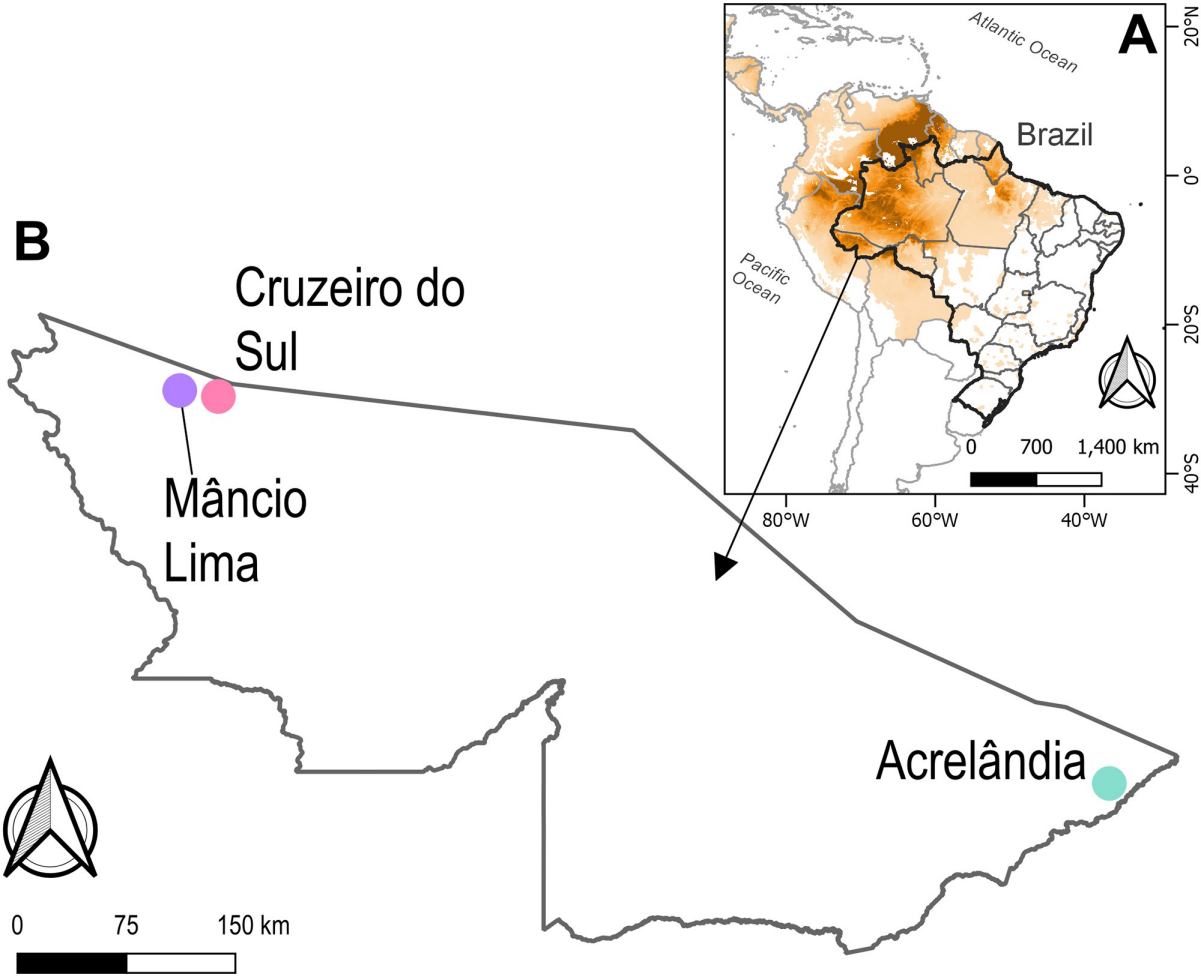
Map of South America showing Acre State, in northwestern Brazil, and the sample collection sites. *A*. Colors represent prevalence rates of *Plasmodium vivax* in 2017 (higher rates in darker shades of orange) as estimated by the Malaria Atlas Project [1]; data available at https://malariaatlas.org/. *B*. Sample collection sites in Acre state in northwestern Brazil. Mâncio Lima (ML) and Cruzeiro do Sul (CS) are located 35 km apart in the upper Juruá Valley region, whereas Acrelândia (AC) is located approximately 700 km southeast of ML and CS. Figure created with QGIS software version 3.12.1, an open source Geographic Information System (GIS) licensed under the GNU General Public License (https://qgis.org/). Publicly available shape files provided from the Brazilian Institute of Geography and Statistics (IBGE) website (https://www.ibge.gov.br/geociencias/downloads-geociencias.html) and GADM maps (https://gadm.org/).

The municipality of Mâncio Lima (ML; population, 18,638) covers a surface area of 4,672 km^2^ in the upper Juruá Valley region, northwestern Brazil (Fig 1). Half of its inhabitants reside in the municipal seat (7°37'20” S, 72°53'32” W), where 48% of the local malaria cases are reportedly acquired. Streams, wetlands, and man-made fish farming ponds are widespread in the town and serve as breeding sites for the primary malaria vector, *Anopheles darlingi* [15]. The annual parasite incidence (API), 521 cases per 1,000 inhabitants in 2017, is the highest for a municipality in Brazil [16]. *Plasmodium vivax* accounts for 84.2% of local malaria cases and *P*. *falciparum* for 14.4%; 1.4% are coinfections with both species [17].

### Sample preparation

Venous blood samples (10–50 ml) were collected between June 2014 and November 2018 from urban residents attending malaria clinics in ML. A single blood sample was obtained from each patient. Samples were filtered through BioR01 Plus leukocyte-depletion devices (Fresenius Kabi, Bad Homburg, Germany) to minimize human DNA contamination prior to genome sequencing [6]. Single-species *P*. *vivax* infection was confirmed by microscopy and quantitative PCR as described [18]. Template DNA was isolated with QIAamp DNA blood kits (Qiagen, Hilden, Germany).

### Whole-genome sequencing

Nextera XT or TrueSeq Nano DNA libraries (Illumina, San Diego, CA) were prepared to generate paired-end short sequence reads (150 bp) on Illumina HiSeq 2500 or HiSeq X platforms. Reads with expected base call accuracy ≥ 99.9% were mapped onto the reference *P*. *vivax* genome PvP01 (PlasmoDB release 35) [19] using the Burrows-Wheeler aligner [20] and the Samtools data processing tool [21]. Single-nucleotide polymorphisms (SNPs) were called using the UnifiedGenotyper tool [22] following the GATK Best Practices (https://software.broadinstitute.org/gatk/best-practices/) and sites with <5× coverage were filtered out. SnpEff [23] was used to identify SNPs mapping to coding sequences (further classified as synonymous or nonsynonymous), introns, and intergenic regions of the PvP01 reference genome. Samples with ≤15% of missing SNP calls were selected for further analyses. We defined the accessible core genome of 21.4 megabases (Mb) that allowed for reliable genotyping calls as described [24] and identified SNPs where an unusually high percentage of samples presented within-sample variation using a previously described strategy [25]. To this end, we plotted the distribution of heteroallelic SNP call rates and identified a conservative cut-off for the tail on the right side to define “hyper-heterozygous SNPs” [25]–those with more heterozygous calls than expected from their allele frequency in the population—that are likely to have been affected by alignment artefacts. We removed a total of 52,955 outlier SNPs with a heterozygosity log score above the cut-off value of 2.2 and the remaining 78,033 SNPs were used to obtain estimates of infection complexity. This was assessed using the within-sample *F* statistic (*F*_WS_) [25], with a threshold of *F*_WS_ > 0.90 (instead of the most usual threshold of *F*_WS_ > 0.95) applied to the core genome to define predominantly single-clone infections. This *F*_WS_ threshold was applied to publicly available *P*. *vivax* genome reads that were processed exactly as described above and shown to correctly classify all samples originally defined as containing single- or multiple-clone infections, based on the most stringent *F*_WS_ threshold of 0.95 used in the original publication [26] (S1 Table). Heteroallelic SNP calls (between 0.01 to 0.05% of all SNPs per sample) were masked prior to data analysis. Finally, we excluded singleton SNPs and those with >5% missing calls across all samples.

### Additional genomic datasets

We reanalyzed raw paired-end whole-genome reads from *P*. *vivax* isolates from two sites in Acre State, Brazil: (a) CS, consisting of 16 samples [27] collected between 2013 and 2016 in the city of Cruzeiro do Sul (7°39′54″S, 72°39′01″W; population, 82,622), 35 km from ML, with an API of 253.5 cases per 1,000 inhabitants in 2017, and (b) AC, consisting of 8 samples [6] collected between 2011 and 2013 in the town of Acrelândia (9°49'31”S, 66°53'11”W; population, 14,366), 700 km southeast of ML, with an API of 6.4 cases per 1,000 inhabitants in 2017 (Fig 1). Fastq files were downloaded from SRA and processed in the same way as our new sequences.

### Data analysis

The ML population of *P*. *vivax* was first assessed for levels of genetic diversity and linkage disequilibrium (LD). We measured genetic diversity using π, the average number of pairwise differences per site. To examine how nucleotide diversity varies across the core genome, we plotted π values as moving averages within 1-kb sliding windows along each chromosome. Pairwise linkage disequilibrium (LD) between SNPs along the same chromosome was measured by *r*^2^ [28], the square of the correlation coefficient between SNP pairs, using VCFtools [29]. To estimate the rate at which LD decays with increasing physical distance between SNPs due to meiotic recombination, *r*^2^ values were binned by distance between SNPs with minor allele frequency > 5% (50-bp windows) and medians within each window were plotted against this distance. The background level of LD was estimated by calculating the median *r*^2^ between pairs of SNPs from different chromosomes.

We further explored the population structure of ML parasites by plotting the distribution of pairwise genetic distances (percentage of SNP mismatches among all SNPs genotyped in each sample pair) [30] and the distance to nearest distribution (distribution of genetic distances between each parasite and its most similar neighbor) [31]. In a panmictic (i.e., randomly mating) population, all individuals can potentially recombine with each other. To test whether local populations of *P*. *vivax*, contrary to panmictic expectations, comprise clusters of highly inbred, closely related parasites, we calculated the *V*_D_/*V*_E_ ratio—the variance of the distribution of empirical pairwise genetic distances between local parasites (*V*_D_) divided by that expected under random association of alleles in a panmictic population (*V*_E_) [32]–as a test for genome-wide LD. To minimize the effect of LD between proximate SNP pairs due to physical linkage on the same chromosome, we generated 1,000 simulated datasets and set to 10 kb the minimal distance (*d*) between SNP pairs randomly sampled along the same chromosome. The total number of randomly chosen SNPs in each simulated dataset (*s*) was set to 1,000. We next examined whether changes in *d* (between 1 bp and 30 kb) and *s* (between 500 and 10,000 SNPs) would affect our ability to detect genome-wide LD. Methodological details are described in the S1 File available online.

We next used a genome-wide identity-by-descent (IBD) approach to characterize the connectivity between parasites within the ML population and across the ML, CS, and AC populations. To this end, we systematically searched for genomic segments that are inferred to have descended from a common ancestor without intervening recombination and estimated the pairwise fraction of shared ancestry between genomes (“IBD fraction”). Analyses were run with the hmmIBD software [33], which implements a hidden Markov model-based approach that accounts for recombination. The recombination rate of *P*. *falciparum*, estimated at 13.5 kb per centiMorgan (cM) [34]–i.e., SNPs separated by 13.5 kb have an average rate of chromosomal crossovers of 1% per generation—was used because no corresponding estimate has been obtained for *P*. *vivax*. Two key parameters were predefined: (a) we set to 25 the maximum number of outcrossed meioses since the most recent common ancestor (“number of generations”) to capture relatively recent gene flow [35], and (b) we set to 0.5 the minimal IBD fraction value used to define highly related parasite pairs that are connected in the network [11]. The 0.5 threshold is approximately equal to the mean relatedness between progeny derived from experimental *P*. *falciparum* crosses [11]. We generated parasite relatedness networks with these predefined parameters and the complete SNP set. We additionally tested whether IBD analyses were robust to changes in the number of SNPs (*s* between 1,000 and 20,000), in the number of generations since the most recent common ancestor (between 10 and 100), and in the minimum IBD fraction required to connect parasites in networks (from 0.5 to 0.2) as described in S1 File.

In addition to IBD, we used principal component analysis (PCA) to explore the genetic affinities between the ML, CS, and AC populations of *P*. *vivax*. While IBD analysis is the approach best suited for describing recent ancestry, PCA would tell more about the underlying, longer-term population structure. Results are shown as briefly discussed in S5 Fig, part of S1 File.

## Results

### Genome-wide diversity in local parasites

We generated the largest collection of genomic data from a single *P*. *vivax* population in the Americas, comprising 59 infections. The 38 isolates from ML whose genome sequences are fully described here harbored predominantly single-clone infections, with *F*_WS_ > 0.90 (64.4% of the 59 analyzed); 22 of them met the most stringent *F*_WS_ threshold of 0.95 [25]. These 38 isolates were sampled in 2014 (n = 14), 2015 (n = 16), 2016 (n = 6) or 2018 (n = 2). They yielded an average read depth of 200×, ranging between 10× and 480× among samples, with a mean of 13 × 10^6^ reads per isolate (S2 Table). On average, 75% (range, 11% to 92%) of the reference PvP01 core genome was mapped with a read depth ≥5×.

After applying stringent quality control filters, we ended up with a catalog of 35,938 high-confidence variable positions that were used in further analyses. Individual ML samples were successfully genotyped at >85% of these variant sites. They had the alternate allele at 768 to 2,988 genotyped sites, with 54.5% of the SNPs located in intergenic regions, 0.3% in introns, and 45.2% in coding regions. Most (59.9%) coding SNPs were nonsynonymous.

**Fig 2:**
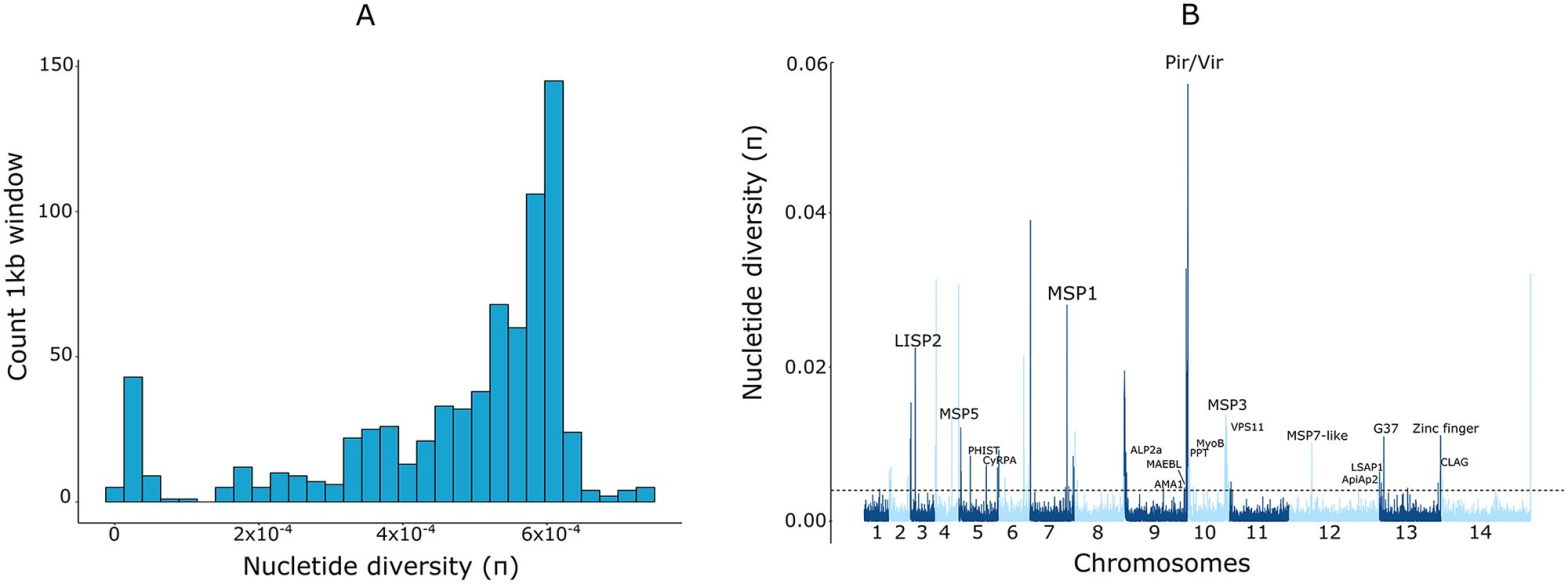
Nucleotide diversity in the Mâncio Lima population of *Plasmodium vivax* in northwestern Brazil. (*A*) Left-skewed distribution of π, the average number of pairwise nucleotide differences per site, with the heavy left tail corresponding to pairwise comparisons within clusters of highly related parasites found in the population. (*B*) Nucleotide diversity π values plotted as moving averages within 1-kb sliding windows along each chromosome. Domains with the top 1% π values are shown above the interrupted horizontal line. The main annotated genes within these high-diversity genome windows are indicated (see also S2 Table for a list of high-diversity domains). The horizontal dotted line indicates the top-1% nucleotide diversity threshold.

The ML population, with its mean π estimated at 4.7 × 10^−4^ (standard deviation, 1.5 × 10^−4^) for the core genome, is slightly less diverse than other local *P*. *vivax* populations from the Americas (range, 5.2 × 10^−4^ to 6.8 × 10^−4^)[4–6], Southeast Asia (5.0 × 10^−4^ to 7.7 × 10^−4^) [4,24,26], and Ethiopia (6.5 × 10^−4^) [26]. The distribution of π values in ML is negatively skewed, with the heavy left tail corresponding to pairwise comparisons within clusters of highly related parasites (Fig 2A). We characterized 189 high-diversity 1-kb windows across the genome that yielded the top 1% π values. Within these windows, we found genes encoding proteins involved in red blood cell invasion, such as the merozoite surface proteins (MSPs) and the apical membrane antigen 1 (AMA1), as well as the AP2 domain transcription factor AP2-O5 [36], the liver-specific protein 2 (LISP2), an early marker of liver stage development [37], and the sexual stage-specific protein G37 [38]. Members of some multigene families, such as *plasmodium helical interspersed subtelomeric* (*phist*) and *cytoadherence linked asexual gene* (*clag*), also mapped to domains with the highest π values (Fig 2B). We note, however, that some diversity spikes near subtelomeric regions may represent artefacts due to poor alignment. S3 Table provides a list of high-diversity genome domains and S1 Dataset provides a list of SNPs putatively associated with antimalarial drug resistance in the ML population.

### Population structure and clusters of genetically related parasites

**Fig 3:**
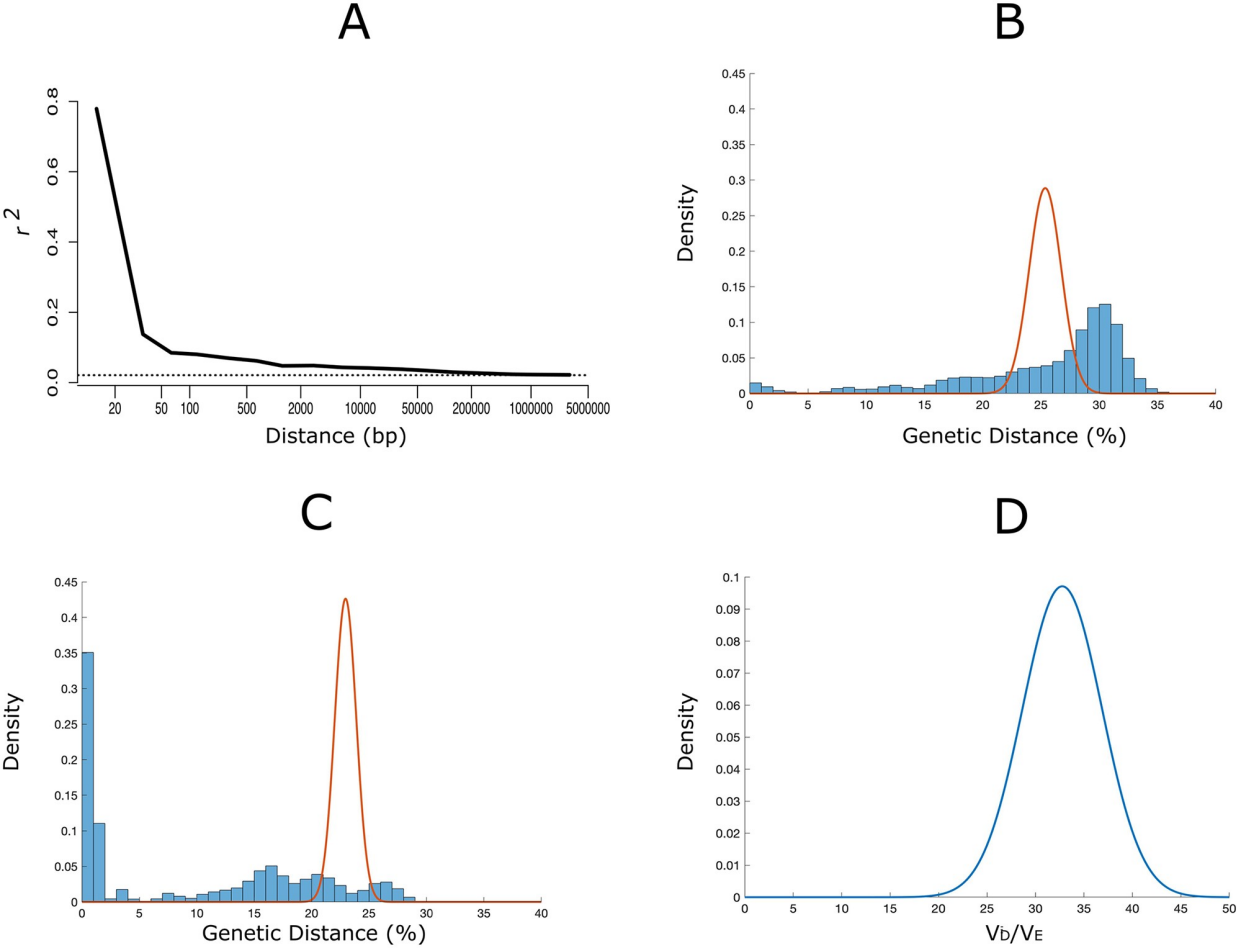
Linkage disequilibrium and population substructure of the Mâncio Lima population of *Plasmodium vivax* in northwestern Brazil. (*A*) Linkage disequilibrium (LD) decay with increasing distance between pairs of SNPs along the chromosome. The horizontal dotted line indicates the background level of LD in the population, given by the median *r*^2^ between pairs of SNPs from different chromosomes. Note that median *r*^2^ values approach the background level within approximately 1.5 kb. (*B*) Empirical distribution of percent genetic distances (percentage of SNP mismatches among all SNPs genotyped in each sample pair [30], shown as blue bars) compared with that expected under random assortment of alleles in a panmictic population (continuous orange line). Note the greater variance of the empirical distribution, which is more spread out along the *x* axis than the expected distribution. (*C*) Empirical distance to nearest distribution (distribution of genetic distances between each parasite and its nearest neighbor [31], shown as blue bars) compared with that expected under random assortment of alleles in a panmictic population (continuous orange line). Note that the empirical distribution is bimodal, with the short-distance distribution to the left corresponding to pairwise comparisons within clusters of highly related parasites. (*D*) Ratio of observed (*V*_D_) to expected (*V*_E_) variances of the distributions shown in panel *B* as a test for genome-wide LD; under panmixia, *V*_D_/*V*_E_ is expected to be equal to 1 [32]. See main text for details.

We examined patterns of genetic distance to test whether the ML population includes discrete clonal or near-clonal lineages maintained in relative genetic isolation within transmission clusters. At the chromosome level, LD in the ML population decays rapidly with increasing distance between SNP pairs and approaches the background level within approximately 1.5 kb (Fig 3A), as expected for organisms undergoing meiotic recombination. At larger scales, however, we found clear evidence of genome-wide LD resulting from population substructure. Indeed, the heavy-tailed distribution of pairwise genetic distances in the ML population clearly deviates from panmictic expectations (Fig 3B) and *V*_D_ largely exceeds *V*_E_ in all simulations with random subsets of SNPs, with *V*_D_/*V*_E_ ratios centered around 32.5 (95% confidence interval, 25.4–41.2) (Fig 3D). These findings are consistent with a highly significant genome-wide LD [32], suggestive of extensive inbreeding in the parasite population. Moreover, the distance-to-nearest distribution is bimodal, with the short-distance distribution to the left arising from comparisons between closely related parasite pairs and the large-distance distribution to the right arising from comparisons between largely unrelated pairs (Fig 3C). Importantly, LD analysis is remarkably robust to changes in the minimum distance between SNP pairs along the same chromosome (*d* varying between 1 bp and 30 kb). However, estimated *V*_D_/*V*_E_ ratios decrease as the number of randomly sampled SNPs (*s*) decreases from 10,000 to 500, although they remain significantly greater than 1 (see S1 File, S1 Fig).

Taken together, these data indicate that the ML population is fragmented into discrete lineages of closely related parasites, with only occasional recombination between them (see below). The barriers to ample recombination between, but not necessarily within, discrete parasite lineages remain undetermined. One hypothesis would be the cocirculation, in the study site, of divergent parasites from varied geographic origins—for example, imported vs. locally acquired infections or newly acquired, sporozoite-induced infections vs. late relapses.

**Fig 4:**
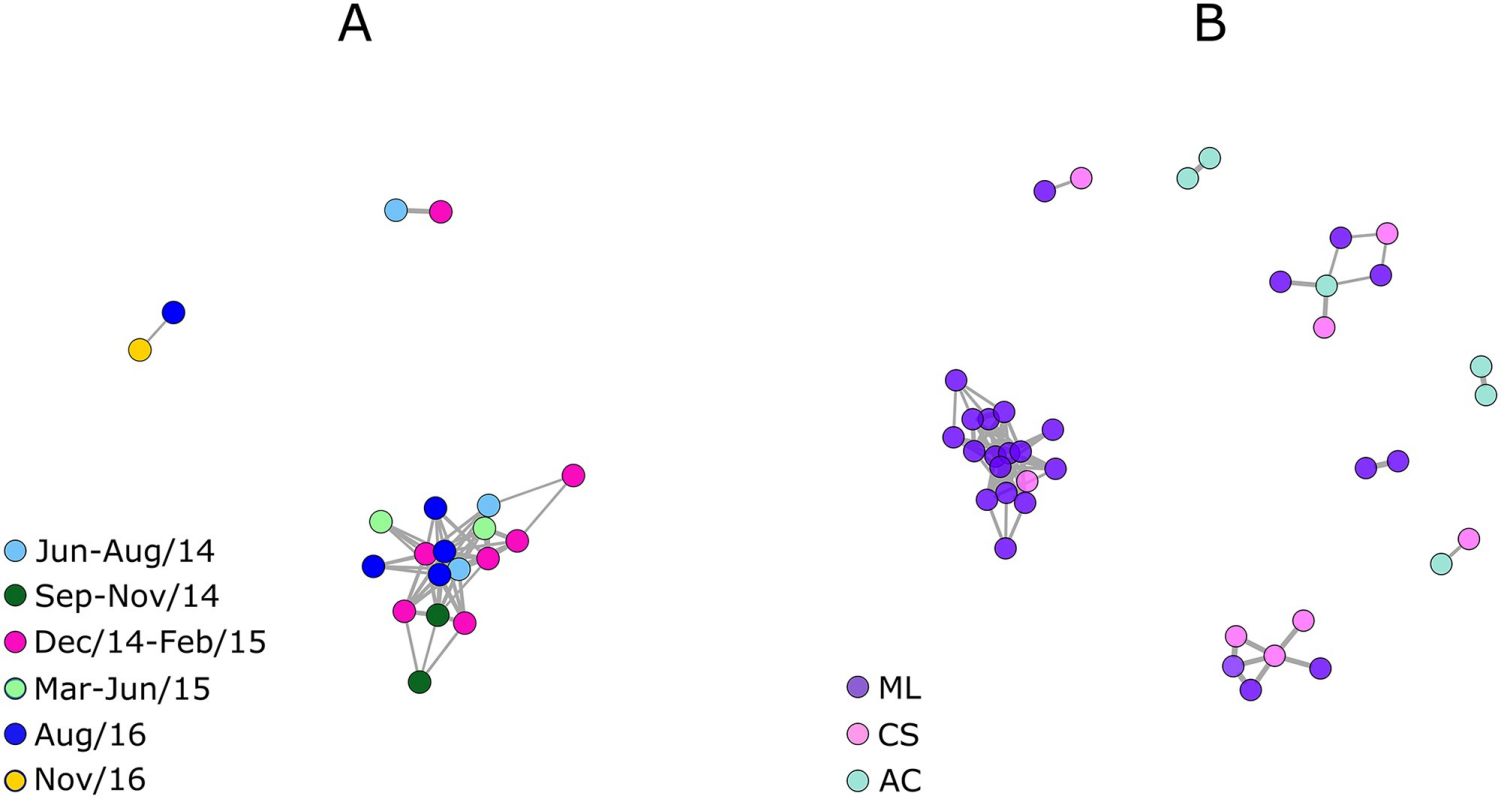
Connectivity networks inferred by identity by descent analysis. Data are shown (*A*) for the *Plasmodium vivax* population of Mâncio Lima (ML) and (*B*) across the populations from ML, Cruzeiro do Sul (CS), and Acrelândia (AC), all in northwestern Brazil. Edges connecting parasite pairs indicate that >50% of their genomes are inferred to have descended from a common ancestor without intervening recombination within the past 25 generations. Isolates that do not share more than 50% of their genome with any other isolate are omitted from the network. Dates of collection of the ML samples are color-coded in panel *A*; note that the large cluster of genetically related isolates shown in panel *A* comprises parasites sampled between June 2014 (light blue) and August 2016 (dark blue).

We used IBD analysis to identify parasites sharing recent ancestry within the ML population (Fig 4A) and across the ML, CS, and AC populations (Fig 4B). We estimated the pairwise fraction of genomes that is inferred to have descended from a common ancestor without intervening recombination within the past 25 generations. The frequency distribution of pairwise IBD fractions in the ML population and across the ML, CS, and AC populations is shown in S1 File, S2 Fig). Pairs of parasites with >50% of shared ancestry are defined as closely related, being connected by edges in the relatedness networks [11]. Of note, the large cluster shown in Fig 4A, which includes 16 of the 38 (42.1%) predominantly single-clone samples sequenced, comprises closely related parasites circulating between June 2014 and August 2016. These findings are consistent with the local propagation of a highly inbred *P*. *vivax* lineage spanning at least 26 months. Relatedness networks of the ML population remain nearly unchanged when the number of generations considered in the analysis increases from 25 to 100 (S1 File, S3 Fig).

**Fig 5:**
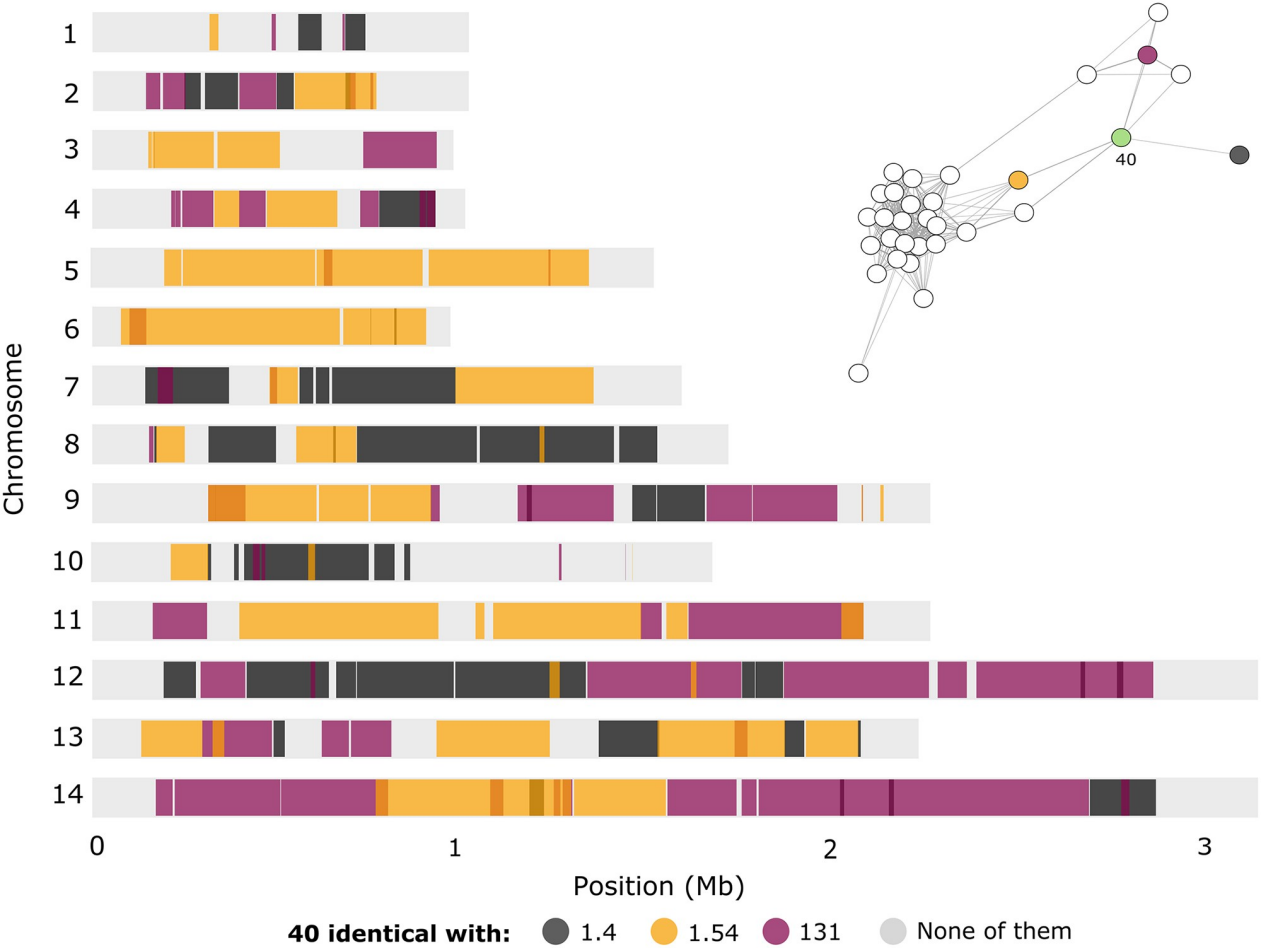
Parasite relatedness and meiotic recombination events in the *Plasmodium vivax* population of Mâncio Lima, northwestern Brazil. An example of genome ancestry sharing consistent with meiotic recombination in this population is highlighted. Isolate 40 shares large sequence blocks with three other parasites: most of chromosomes 5 and 6 are shared with isolate 1.54, chromosome 12 shares sequence blocks mostly with isolates 1.4 and 131, chromosome 7 shares sequence blocks with isolates 1.4 and 1.54, chromosome 11 shares sequence blocks with isolates 1.54 and 131, and chromosome 13 shares sequence blocks with all three isolates. The inset in the right upper corner shows a relatedness network similar to that depicted in Fig 4A but drawn with an identity-by-descent fraction threshold set to 0.2. Note that isolate 40 (green node) bridges the gap between the large cluster of parasites to the left and the smaller cluster of parasites to the right.

By changing the IBD fraction threshold from 0.5 to 0.2 we explore more distant relatedness (S1 File, S3 Fig) that allow us to identify examples of occasional recombination events between lineages [8]. Fig 5 illustrates one such example: isolate 40, sampled in 2016, serves as a node that connects clusters of unrelated parasites (Fig 5 inset). Its chromosomes share sequence blocks with isolates 1.4 (sampled in 2014), 1.54 (sampled in 2015), and 131 (sampled in 2016). Consistent with its position in the relatedness network, isolate 40 also shares sequence blocks with other putative relatives within these clusters of related parasites (S1 File, S4 Fig). Because the proportion of shared ancestry between isolate 40 and each of its putative relatives is below 0.5, their connections were missed in the network depicted in Fig 4A but are revealed by the less stringent analysis shown in Fig 5 inset.

To test whether patterns of genetic connectivity between ML parasites could be retrieved with low-coverage genome sequencing data, we used random subsets of 20,000, 10,000 or 1,000 SNPs to generate relatedness networks and counted the number of edges connecting parasites in 1,000 network replicates. We found that the average number of connections drawn between parasites changes relatively little when curtailed SNP sets are used in IBD analyses with the same parameters as in our main analysis (IBD fraction threshold = 0.5 and number of generations = 25; S1 File, S5 Fig).

### Gene flow and regional spread of *Plasmodium vivax*

Fig 4B shows instances of shared ancestry between parasites in close geographic proximity, from ML and CS, in addition to two examples of shared ancestry between them and the AC population. These findings imply gene flow between ML and CS, the nearby city in the Juruá Valley with a two-fold lower API. Moreover, they are consistent with *P*. *vivax* transmission pathways that originate from ML and CS and reach AC, the site with the lowest API in our analysis, about 700 km east of the Juruá Valley.

## Discussion

The decreasing cost and increasing efficiency of next-generation sequencing have stimulated the analysis of genetic relatedness of pathogens to identify transmission networks [30,39]. Quantifying gene flow between different transmission pockets is of great importance to eliminate residual malaria [10], but methods for transmission network reconstruction developed for rapidly mutating pathogens such as viruses and bacteria do not necessarily apply to malaria parasites [9]. Here we leverage the potential of genome-wide IBD analysis to map the circulation of *P*. *vivax* lineages in the main malaria hotspot of Brazil.

The forces and mechanisms that promote genetic diversity and recombination in *P*. *vivax* remain poorly understood [40]. Here we show that highly inbred parasite lineages propagate in this hotspot but paradoxically present key features of outbreeding organisms. First, natural infections often comprise multiple clones. For example, we found evidence for two or more co-infecting clones in 35.6% or 62.7% of the ML isolates sequenced, depending on whether the less stringent (*F*_WS_ >0.90) or more stringent (*F*_WS_ >0.95) criterion is applied to define a multiplicity of infection greater than one. As a consequence, genetically unrelated gametocytes may be taken during a mosquito blood meal and recombine in the vector midgut. Second, the average genome-wide diversity in the ML population is only slightly lower than levels observed in most *P*. *vivax* populations worldwide [4–6,24,26]. Indeed, *P*. *vivax* parasites in ML are as diverse as predominantly outcrossing *P*. *falciparum* populations e.g. from Senegal (π = 4.5 × 10^−4^; n = 25) [7], the China-Myanmar border (π = 4.6 × 10^−4^, n = 34), the Thai-Cambodia border (π = 3.5 × 10^−4^, n = 56), and the Thai-Myanmar border (π = 3.9 × 10^−4^, n = 40) [41]. We suggest that the relatively high population-level genetic diversity in ML and other substructured populations results from the cocirculation of multiple, relatively distant inbred lineages.

High inbreeding appears to be a common feature of some *P*. *falciparum* populations in Africa and Southeast Asia. For example, discrete *P*. *falciparum* lineages may persist across multiple years in Thiès, a site approaching malaria elimination in Senegal [8]. In addition, closely related parasites can be recovered over large geographic distances from high-endemicity sites in the Democratic Republic of the Congo [42] and low-endemicity sites across Southeast Asia [12]. Here we characterize discrete *P*. *vivax* lineages that remain stable across time and space in one of the areas with the highest malaria transmission in the Americas. Relapses can account for some clonal persistence, because *P*. *vivax* strains are repeatedly reintroduced in the population as hypnozoites reactivate [40]. However, despite the ample opportunities for meiotic recombination and outcrossing between lineages, we find a strong genome-wide LD in the ML population. The population substructure described here is reminiscent of the Wahlund effect, with separate lineages living in sympatry but unable to recombine. Substructure may have different origins. For example, imported lineages circulating in the town may not originate substantial onward transmission and, if so, there could effectively be isolation even with sympatric sampling. Strikingly, a single highly inbred lineage comprises almost half of isolates sampled in ML over two years. How discrete lineages of *P*. *vivax* persist nearly unchanged over time remains open to speculation and evolutionary forces that actively restrain recombination have been hypothesized [43]. Population bottlenecks following the emergence of antimalarial drug resistance could be an explanation, as they contribute to increased parasite relatedness and can shape the regional population structure of malaria parasites [5,12,35]. However, we argue that drug-associated selective sweeps are very unlikely to have played a major role in the Juruá Valley hotspot, where resistance to chloroquine, the first-line antimalarial drug used to treat *P*. *vivax* infections in the Americas, remains very rare [18,44].

Patterns of shared ancestry are consistent with gene flow between ML and the nearby city of CS, where massive malaria outbreaks have occurred in the mid-2000 [45] and local transmission remains high [16]. The extensive rural-urban mobility across the Amazon [46] continuously introduces parasites into densely populated and receptive urban spaces, leading to explosive epidemics [45] or endemic urban malaria transmission [47]. Although the directionality of gene flow cannot be determined by IBD analysis, the finding of shared ancestry between *P*. *vivax* isolates from high-transmission ML and low-transmission AC, >700 km apart from each other, suggests the likely contribution of parasites originating from the ML hotspot to ongoing residual malaria transmission in vast areas of northwestern Brazil [14].

Genome sequencing remains challenging for *P*. *vivax* because of the low parasitemias in natural infections, the large amount of human DNA contamination in clinical samples, and the lack of practical methods for long-term parasite propagation in vitro [48]. Importantly, our results indicate that as few as 500 SNPs would be enough to identify genome-wide LD and 1,000 SNPs would resolve major clustering patterns in the ML population, although the minimum number of SNPs required for robust IBD analysis remains to be determined for different populations [42,49]. Expanding parasite populations, for example, which have disproportionally more rare alleles, are likely to require more SNPs to recover IBD relationships accurately. We suggest, however, that relatively low-coverage genome sequencing or genotyping panels of hundreds of markers, each with multiple SNPs, may suffice to elucidate key aspects of *P*. *vivax* epidemiology in most endemic settings.

This study has some limitations. First, we have analyzed a limited number of parasite sequences from three locations and key nodes in relatedness networks have surely remained unsampled. We are currently expanding our sample collection efforts to a wide range of geographic sites across northwestern Brazil. Second, the present analysis is limited to *P*. *vivax*, the dominant malaria parasite in Brazil [14]. Identifying the main *P*. *falciparum* transmission pathways in this country remains a matter for future population genomic investigation. This is particularly important in the regional context, given that nearly 40% of *P*. *falciparum* malaria cases in Brazil originate in the upper Juruá Valley hotspot.

We conclude that highly inbred *P*. *vivax* lineages spread over time and large geographic distances in a major malaria hotspot in northwestern Brazil. Genomic epidemiology approaches may help to map source populations and prioritize areas for targeted control interventions to eliminate residual *P*. *vivax* transmission in the Amazon and similar endemic settings worldwide.

## Supporting information

S1 File Supplementary methods and S1 Fig, S2 Fig, S3 Fig, S4 Fig, and S5 Fig. (DOCX)

S1 Table Comparison of originally calculated *F*_WS_ estimates for Ethiopian genomes of *P*. *vivax* (Alburn et al., 2019) and those obtained for the same samples after applying to raw reads the SNP call and filtering processes described in the main text. (XLSX)

S2 Table Newly generated *Plasmodium vivax* genome sequences from Mâncio Lima, northwestern Brazil, and their SRA accession numbers. (XLSX)

S3 Table Complete list of highly diverse genome domains (1-kb windows) in the Mâncio Lima population of *Plasmodium vivax*, northwestern Brazil, with genome positions, identifiers in the PVP01 reference genome and associated π values. (XLSX)

S1 Dataset SNPs in select genes putatively associated with antimalarial drug resistance in *Plasmodium vivax* genome sequences from Mâncio Lima, northwestern Brazil. (XLSX)
