# Supplementary material for: Population genomics reveals the expansion of highly inbred Plasmodium vivax lineages in the main malaria hotspot of Brazil
Source: PLoS Negl Trop Dis. 2020 Oct 28;14(10):e0008808. doi: 10.1371/journal.pntd.0008808 (PMC7592762; doi:10.1371/journal.pntd.0008808)
Supplement: S1 File — (DOCX) [file pntd.0008808.s001.docx]

**S1 File**

**Population genomics reveals the expansion of highly inbred *Plasmodium vivax* lineages in the main malaria hotspot of Brazil**

de Oliveira *et al*.

**Supplementary Methods and Results**

**Genome-wide linkage disequilibrium (LD)**

One popular way to test whether populations of microorganisms are structured into clonal or near-clonal lineages that remain genetically isolated from each other is based on the variance of the distribution of pairwise differences (*V*_D_) observed among isolates that have been genotyped at multiple loci. *V*_D_ is compared to the variance *V*_E_ expected under the null hypothesis of panmixia, with all loci in complete linkage equilibrium. *V*_D_ is expected to increase, relative to *V*_E_, in populations comprising discrete clonal or near-clonal lineages [*1*]. The distribution of *V*_E_ can be estimated by randomly reshuffling the original alleles among genomes so that the numbers of alleles and their frequencies at individual loci are exactly the same in each replicate as in the original sample [*2*]. Tests for multilocus LD based on comparisons between *V*_D_ and *V*_E_ were introduced four decades ago [*3*] and have been extensively applied to the population genetics of bacteria [*4,5*] and malaria parasites (e.g., [*6*]). Importantly, these tests assume independent segregation of genetic markers – whenever two or more loci are typed along the same chromosome, they must be distant from each other in order to allow for frequent meiotic recombination events between them. Here we extend to genome-wide sequence data the comparison between *V*_D_ and *V*_E_ as a test for LD and propose simple ways to circumvent key obstacles.

First, we have different numbers of SNPs in each pairwise comparison, because genome sequencing coverage varies among isolates and only a relatively small proportion of SNPs are genotyped in all samples. We thus consider the proportion (instead of the absolute number) of pairwise SNP mismatches – or “percent genetic distance” – in our genome-wide LD analyses and redefine *V*_D_ and *V*_E_ as the variance of the observed and expected distributions of percent genetic distances, respectively. We also computed the observed and expected percent genetic distances between each parasite and its nearest neighbor, or “distance to nearest”, for the same datasets. We note that populations substructured into lineages of nearly identical parasites typically display bimodal distance to nearest distributions [*7*].

Second, genome-wide sequencing identifies many SNP pairs that are adjacent to each other on the same chromosome and thus violate the assumption of independent segregation of alleles. In other words, SNPs may be at LD simply because of proximity or physical linkage. To minimize the overall effect of LD between proximate SNP pairs, we imposed a minimal distance *d* (either 1 kb, 10 kb or 50 kb) between SNPs randomly sampled along the same chromosome. For comparison purposes, we also generated datasets with randomly sampled SNPs with no restrictions regarding adjacent SNPs (minimum distance *d* =1 bp).

To test the null hypothesis that *V*_D_/*V*_E_ = 1, as expected under panmixia [*2*], we estimated the distribution of *V*_D_/*V*_E_ in 1,000 simulated datasets with predefined *d* values. We note, however, that the number of available genetic markers (subset $S$) is drastically reduced by increasing $d$. We thus sought to determine the minimum SNP genotyping density required for detecting genome-wide LD. Based on 1,000 iterations ($n_{s}$), we varied between 500 and 10,000 the number $s$ of randomly select SNPs (subset $S$) constrained to $d$ and retested the null hypothesis with these curtailed datasets. We note that, although replicates comprised reduced numbers of SNPs, signal from the majority of the SNPs in the dataset was likely to be explored in our analyses due to the randomization and the high number of replicates.

All simulations described above were carried out using MATLAB according to Algorithm 1.

**Algorithm 1:** Generating simulated datasets with different numbers of randomly selected SNPs (*s*) and different minimal distances between SNP pairs (*d*).

Input:

- sample data matrix $M\in R^{n x m}$, where $n$ is the number of samples and $m$ the number of SNPs
- number $s$ of SNPs of the subset $S$ ($s\leq m$)
- minimal distance between SNP pairs $d$

Repeat for $i=1,\ldots,n_{s}$

1. Compute the matrix $D_{i}\in R^{n x s}$ by randomly selecting a subset $S$ of SNPs constrained to $d$
2. Compute the matrix $E_{i}\in R^{n x s}$ by permuting the elements of each column (alleles) of $D_{i}$
3. Compute the pairwise genetic distance matrices $\Delta_{D,i},\Delta_{E,i}\in R^{n x n}$ of $D_{i}$ and $E_{i}$, respectively
4. Compute the nearest neighbor distance vector $N_{D,i}, N_{E,i}\in R^{n x 1}$ of $D_{i}$ and $E_{i}$ by selecting the minimal value of each row out of the main diagonal of $\Delta_{D,i}$ and $\Delta_{E,i}$, respectively
5. Compute the variance $V_{D,i}$ and $V_{E,i}$ of the elements above the main diagonal of $\Delta_{D,i}$ and $\Delta_{E,i}$, respectively, and compute the ratio $V_{D,i}/V_{E,i}$
6. Store $\Delta_{D,i},\Delta_{E,i}, N_{D,i}, N_{E,i}$ and $V_{D,i}/V_{E,i}$

Output:

- $\Delta_{D,i},\Delta_{E,i}$, $i=1,\ldots,n_{s}$.
- $N_{D,i}, N_{E,i}$, $i=1,\ldots,n_{s}$.
- $V_{D,i}/V_{E,i}$, $i=1,\ldots,n_{s}$.

Similar *V*_D_/*V*_E_ results were obtained when varying *d* between 1 bp and 30 kb, but *V*_E_ estimates increase substantially with decreasing s. We note, however, that although the ratio *V*_D_/*V*_E_ decreases when fewer SNPs are considered (lower $s$), *V*_D_/*V*_E_ distributions do not include the value 1, rejecting the null hypothesis at <1% level of significance.

**Identity-by-descent (IBD) clustering**

We assume that parasites sampled from different hosts sharing recent ancestry are very likely to part of the same transmission network [*8*]. Two key parameters to consider in IBD analysis are: (a) the maximum number of outcrossed meioses since the most recent common ancestor (“number of generations”) used to define recent ancestry shared by a sample pair and (b) the critical value of genome-wide IBD fraction used to connect highly related samples in networks [*9*]. S2 Fig shows the frequency distribution of pairwise IBD fractions in the ML population and across the ML, CS, and AC populations obtained with 25 generations.

We sought to assess how sensitive is IBD-based clustering to changes in these parameters. To this end, we compared relatedness networks obtained with three different numbers of generations (10, 25 or 100) and two different critical values of IBD fraction (0.5 or 0.2). We found the same numbers of parasites (nodes) within networks from IBD analyses with 25 and 100 generations, although the total number of connections (edges) between them will differ. However, very little connectivity was found in simulations with 10 generations (S3 Fig). Not surprisingly, more nodes and edges are found in networks built with a more relaxed definition of genetic relatedness (IBD fraction ≥ 0.2; S3 Fig).

We describe in the main text how networks with a less strict definition of relatedness (IBD fraction ≥ 0.2) can help to reveal putative examples of occasional recombination events between unrelated parasite lineages. Here we provide another example that involves isolate 40, sampled in 2016, and isolates 1.4, 2.4, and 1.45, all sampled in 2014 (S4 Fig).

To test whether genetic connectivity patterns could be retrieved with low-coverage genome sequencing data, we used random subsets with reduced numbers of SNPs (*s* = 1,000, 10,000 or 20,000) sampled from across the genome. Because IBD takes into account expected recombination rates between SNPs at different map distances along the chromosomes [*9*], we did not impose any minimum distance *d* between randomly selected SNP pairs in this analysis. We counted the number of edges connecting parasites in 1,000 replicates of relatedness networks generated with reduced SNP sets for each minimum IBD fraction value (0.5 or 0.2), and for each number of generations (25 or 100) and compared these results with the number of edges obtained with the full set of 35,938 SNPs. We do not show results for 10 generations because very few edges were found in the original IBD analysis (all SNPs considered) with 10 generations (S3 Fig). We found that reducing *s* does not necessarily reduce the number of edges connecting parasites in IBD analyses that apply the most stringent critical IBD fraction value (0.5). In fact, the number of edges may even increase when using reduced SNP sets, as shown for the simulations with 100 generations, and we currently do not have an explanation for this, other than stochastic variation, to offer. In the less stringent analysis (IBD fraction threshold = 02), however, the number of edges is substantially reduced with s = 1,000 (S5 Fig).

**Principal component analysis**

In addition to IBD, we used principal component analysis (PCA) to explore the genetic affinities between the ML, CS, and AC populations of *P. vivax*. While IBD analysis is the approach best suited for describing recent ancestry, PCA would tell more about the underlying, longer-term population structure. We note, however, that PCA may be affected by clusters of highly correlated SNPs across the genome. The PCA plot did not prove very informative regarding the clustering patterns observed within and across populations, except for the cluster of ML and CS samples off to the right in PC2 (S5 Fig), which corresponds to one of the IBD clusters in Fig 4B (see main text).


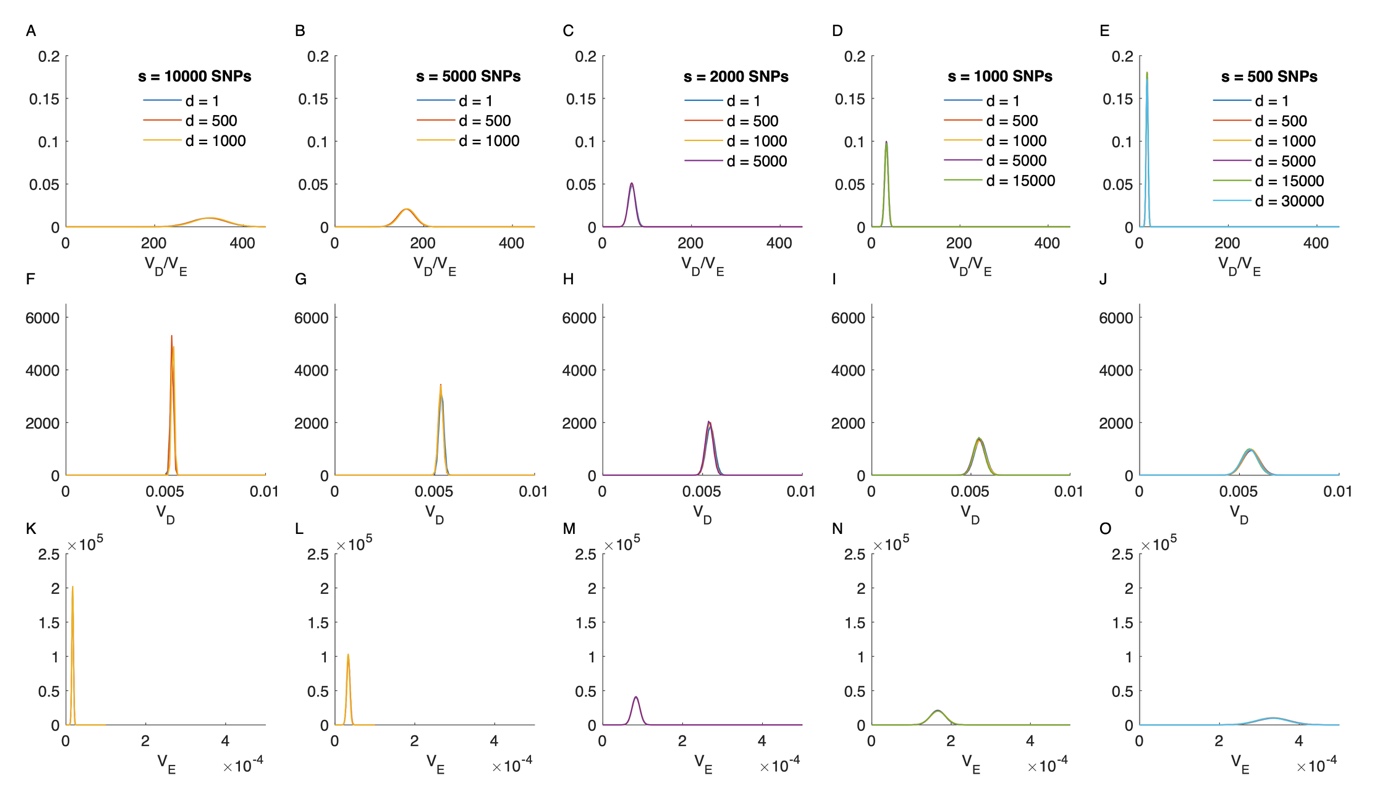


**S1 Fig.** Distribution of *V*_D_/*V*_E_ ratios in 1,000 simulated datasets with SNP distances (*d*) ranging from 1 bp to 30 kb and SNP counts (*s*) varying between 1000 and 10,000. Note that estimates are sensitive to changes in *s* (although in all cases *V*_D_/*V*_E_ >>1; compare distributions across panels A-E) but not to changes in *d* (compare distributions within panels). In fact, *V*_D_ estimates remain nearly unchanged when *s* decreases (panels F-J), but *V*_E_ estimates increase substantially with decreasing *s* (panels K-O).

**
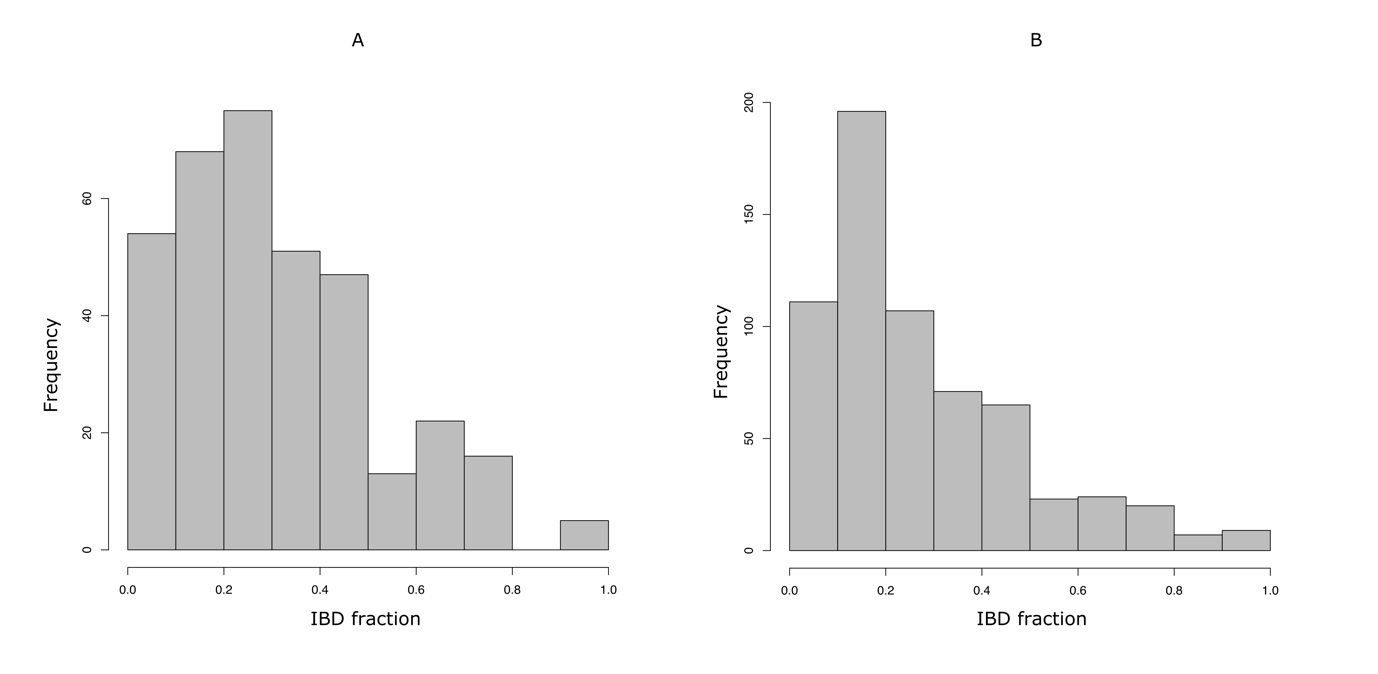
**

**S2 Fig**. Frequency distribution of pairwise identity-by-descent (IBD) fractions in the ML population (*A*) and across the ML, CS, and AC populations *(B*) obtained with 25 generations.


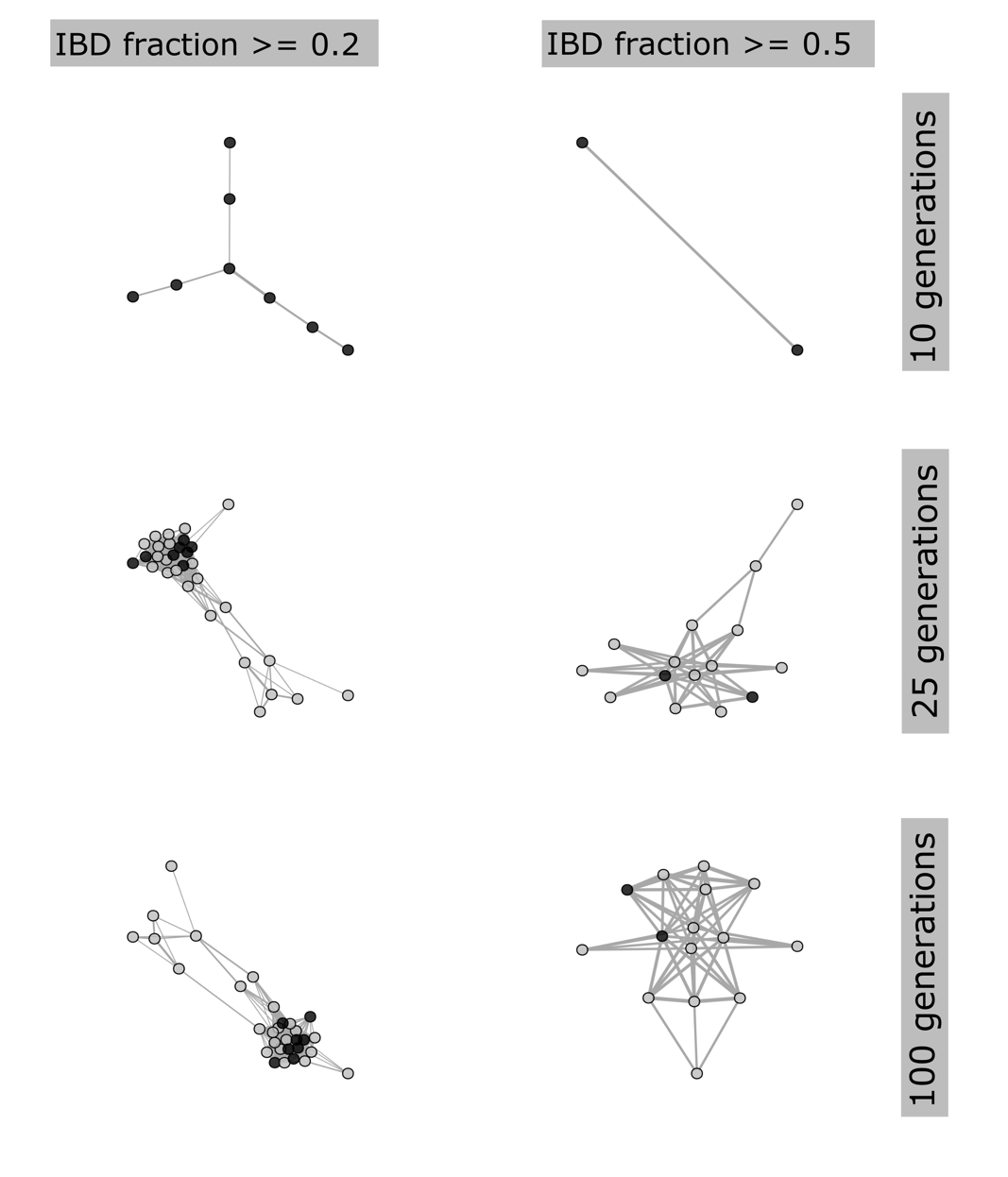


**S3 Fig.** Networks of parasite relatedness in the ML population based on IBD analysis of whole-genome data considering different numbers of generations (10, 25, or 100). Each node identifies a unique parasite and an edge is drawn between two isolates with IBD fractions ≥ 0.2 (left panel) or ≥ 0.5 (right panel). Note that relatively few parasites (represented by black dots) are connected in the analyses considering only 10 generations; these are the sample pairs that presumably share the most recent ancestry. The networks obtained with 25 and 100 generations have the same numbers of nodes, but more edges are seen with 100 generations.


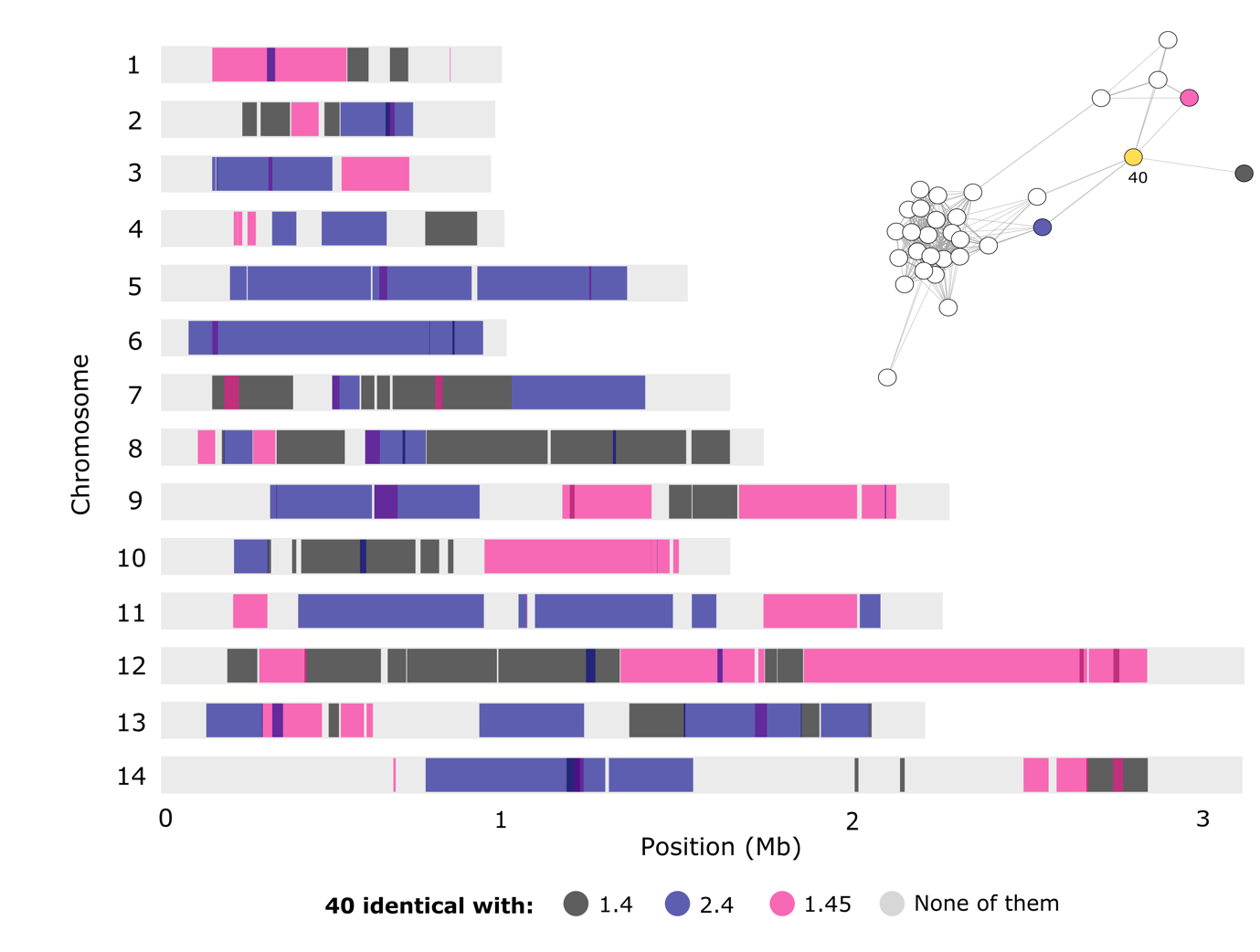


**S4 Fig.** An additional example of occasional meiotic recombination in the *Plasmodium vivax* population of Mâncio Lima, northwestern Brazil. Comparison of Isolate 40 with three other parasites reveals several blocks of shared sequence: most of chromosomes 5 and 6 are shared with isolate 2.4, chromosome 12 shares sequence blocks with isolates 1.4 and 1.45, and chromosomes 7 and 13 share sequence blocks with all three isolates. The inset in the right upper corner locates isolates 40, 1.4, 1.45, and 2.4 in a relatedness network drawn with an identity-by-descent fraction threshold set to 0.2.


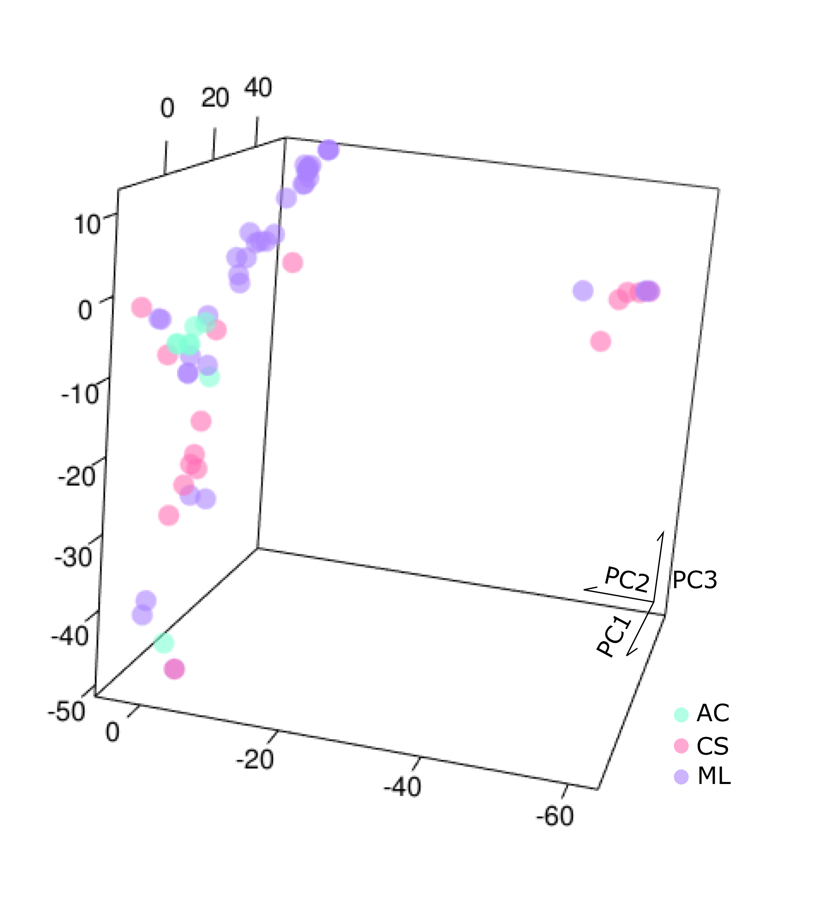


**S5 Fig.** Genetic relatedness among *Plasmodium vivax* isolates from Mâncio Lima (ML), Cruzeiro do Sul (CS), and Acrelândia (AC), all in northwestern Brazil, as shown in a three-eigenvector PCA plot using genome-wide SNP variation data. Note that the cluster of ML and CS samples off to the right in PC2 corresponds to one of the IBD clusters in Fig 4B (see the main text).


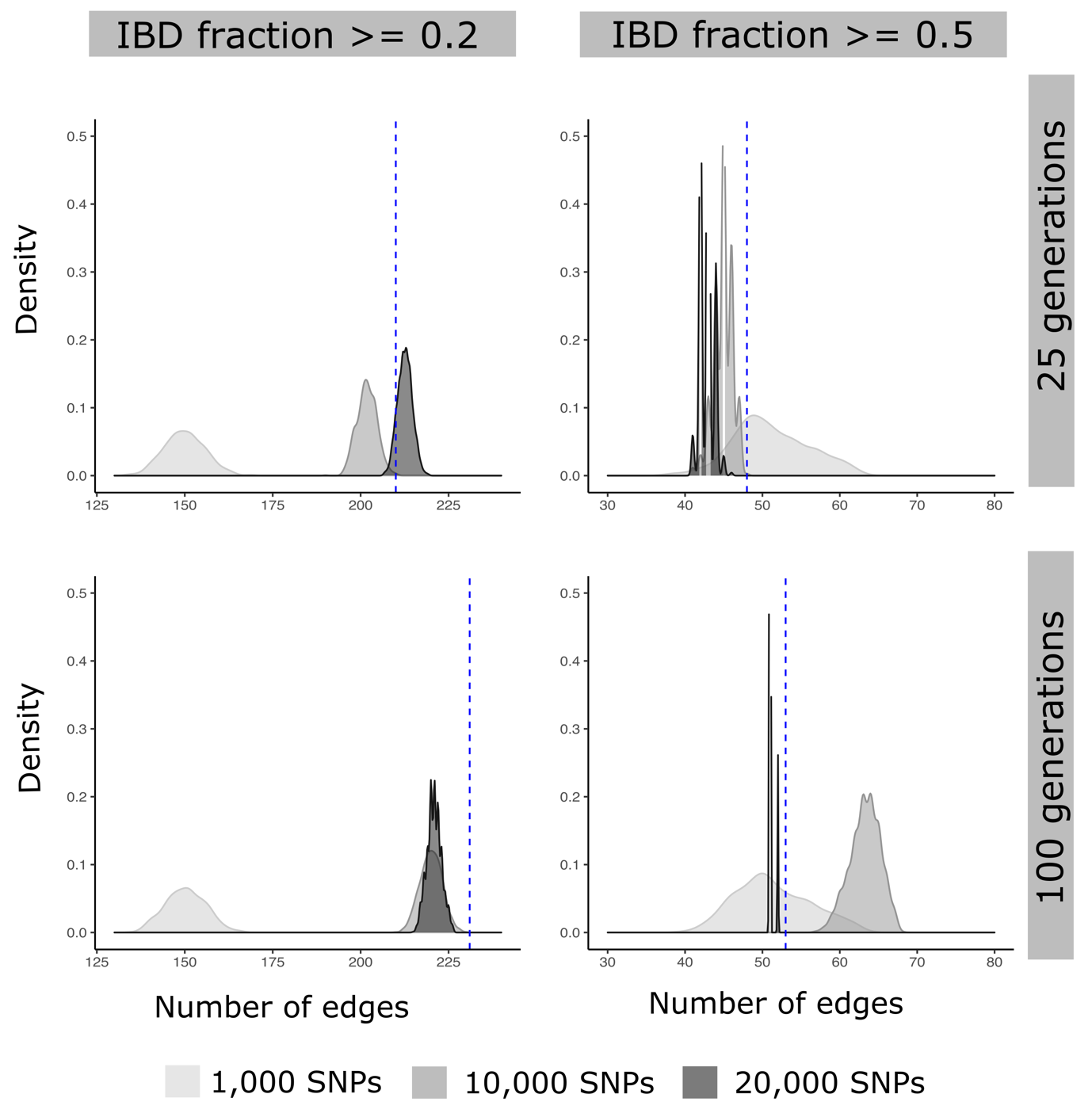


**S5 Fig.** Number of edges in relatedness networks obtained with curtailed SNP sets and different critical values of IBD fraction. We generated 1,000 simulated datasets with the number of randomly sampled SNPs *s* set to either 1,000, 10,000 or 20,000 and counted the number of edges in networks built with each curtailed dataset. The number of edges obtained with all 35,938 SNPs (as in S2 Fig) is represented by the vertical blue line.

**Supplementary References**

1. Hudson RR. Analytical results concerning linkage disequilibrium in models with genetic transformation and recombination. J Evol Biol 1994; 7:535-548.

2. Haubold B, Travisano M, Rainey PB, Hudson RR. Detecting linkage disequilibrium in bacterial populations. Genetics 1998;150:1341-1348.

3. Brown AHD, Feldman MW, Nevo E. Multilocus structure of natural populations of *Hordeum spontaneum*. Genetics 1980;96:523-536.

4. Whittam TS, Ochman H, Selander RK. Multilocus genetic structure in natural populations of *Escherichia coli*. Proc Natl Acad Sci USA 1993;80: 1751-1755.

5. Maynard Smith J, Smith NH, Dowson CG, Spratt BG. How clonal are bacteria? Proc Natl Acad Sci USA 1993;90: 4384-4388.

6. Anderson TJ, Haubold B, Williams JT, et al. Microsatellite markers reveal a spectrum of population structures in the malaria parasite *Plasmodium falciparum*. Mol Biol Evol. 2000;17:1467-1482.

7. Nouri N, Kleinstein SH. A spectral clustering-based method for identifying clones from high-throughput B cell repertoire sequencing data. Bioinformatics. 2018;34:i341-i349.

8. Taylor AR, Schaffner SF, Cerqueira GC, et al. Quantifying connectivity between local *Plasmodium falciparum* malaria parasite populations using identity by descent. PLoS Genet. 2017;13:e1007065.

9. Schaffner SF, Taylor AR, Wong W, Wirth DF, Neafsey DE. hmmIBD: software to infer pairwise identity by descent between haploid genotypes. Malar J. 2018;17:196.
